# Gibberellic Acid and Silicon Ameliorate NaCl Toxicity in *Brassica juncea:* Possible Involvement of Antioxidant System and Ascorbate-Glutathione Cycle

**DOI:** 10.3390/plants12061210

**Published:** 2023-03-07

**Authors:** Pravej Alam, Thamer Al Balawi, Sami Ullah Qadir, Parvaiz Ahmad

**Affiliations:** 1 Department of Biology, College of Science and Humanities, Prince Sattam Bin Abdulaziz University, Al-Kharj 11942, Saudi Arabia; 2 Department of Environmental Sciences Government, College for Women, Udhampur 182101, India; 3 Department of Botany, Government Degree College, Jammu and Kashmir, Pulwama 192301, India

**Keywords:** gibberellic acid, silicon, salinity, stress, *B. juncea*, antioxidants

## Abstract

This work was carried out to observe the combined impact of exogenous applications of Gibberellic acid (GA_3_) and Silicon (Si) on *Brassica juncea* under salt (NaCl) stress. Application of GA_3_ and Si enhanced the antioxidant enzyme activities of (APX, CAT, GR, SOD) in *B. juncea* seedlings under NaCl toxicity. The exogenous Si application decreased Na^+^ uptake and enhanced the K^+^ and Ca^2+^ in salt stressed *B. juncea*. Moreover, chlorophyll-*a* (*Chl-a*), Chlorophyll-*b* (*Chl-b*), total chlorophyll (*T-Chl*), carotenoids and relative water content (RWC) in the leaves declined under salt stress, which were ameorialated after GA_3_ and Si supplementation individually and in combination. Further, the introduction of Si to NaCl treated *B. juncea* help in alleviating the negative effects of NaCl toxicity on biomass and biochemical activities. The levels of hydrogen peroxide (H_2_O_2_) increase significantly with NaCl treatments, subsequently resulting in enhanced peroxidation of membrane lipids (MDA) and electrolyte leakage (EL). The reduced levels of H_2_O_2_ and enhanced antioxidantactivities in Si and GA_3_ supplemented plants demonstrated the stress mitigating efficiency. In conclusion, it was observed that Si and GA_3_ application alleviated NaCl toxicity in *B. juncea* plants through enhanced production of different osmolytes and an antioxidant defence mechanism.

## 1. Introduction

Salinity is one of the most severe abiotic stress factors limiting plant growth and yield by hampering photosynthetic and metabolic activities [1,2]. Plants’ exposure to higher concentration of salts in agricultural soils are an issue of significant concern, particularly in arid and semi-arid climatic regions [3]. According to reports, about 45 to 800 million hectares [1] of the worlds irrigated land area is salt affected [3,4,5]. To fulfil the food demands of increasing populations, the productivity of land resources need to be improved, which can be achieved through the use of less suitable or marginal soils, such as those with a high concentration of salts, deficient in available nutrients, low water holding capacity and slightly polluted soils into cultivation [2]. Soil salinity adversely affects food crop production [1] in terms of both quality and quantity by high osmotic potential and the toxicity of different metal ions [6,7]. Increased soil salinity causes a significant decrease in different aspects of biomasses including root and shoot fresh weights [8]. In saline environments, plant flora is exposed to two different problems. In the first instance, the concentration of higher salts in soils lowers the osmotic potential of the soil solution, leading to a reduction in water uptake in plants, and secondly improved translocation of Na^+^ and Cl^−^ ions causes the interruption of essential mineral nutrients uptake [9], imputes plant toxicity and results in the lowering of agricultural productivity [10].

To advance THE plant productivity raised in marginal soils, the role of silicon (Si) can no longer be neglected. Being the second most abundant element in the earth’s crust and being associated with improving the salinity stress in plants by increasing salt resistance, a decrease in the translocation of toxic elements thereby increases biomass yield [11]. Si plays a prominent role in the uptake, distribution and functionality of different macro and micronutrients. Among macro nutrients nitrogen (N), phosphorous (P), potassium (K), calcium (Ca) and magnesium (Mg) are influenced in different ways [11,12]. In the family of micronutrients, boron (B) and manganese (Mn) are highly influenced by means of Si. Furthermore, Si also influences the uptake of elements such as iron (Fe), Aluminium (Al) and Zinc (Zn) [13,14]. The application of Si has been demonstrated to be a beneficial element for the growth and development of various plants and it also alleviates various stresses, including nutrient imbalance in crop plants [11,12,13,14].

Among the class of different growth regulators, Gibberellic acid (GA_3_) is known to induce many important physiological responses in different plant species [5,15,16]. In maintains the growth retardation of plants due to the excessive concentration of salts and advanced effectiveness of the exogenic application of GA_3_ on various physiological, biochemical and morphological events. It can be inferred that the exogenous introduction of GA_3_ is suitable to alleviate salt stress with its effectiveness being more prominent in salt tolerant species [5,17]. GA_3_ mitigates salt stress by partially increasing plant metabolic processes. GA_3_ also helps in the stimulation of different enzymatic and non-enzymatic antioxidants and the accumulation of osmolytes in plants [18]. The application of exogenous GA_3_ proliferates plant development by leading to the intensification of amino acid synthesis of hydrolytic enzymes as a prerequisite for the breakdown of endospermic starch when plant seeds renovate development at the seed propagation stage [5]. The aim of the present work is to study the role of combined GA_3_ and Si in mitigating NaCl toxicity in mustard through the modulation of biochemical pathways and the activities of enzymatic antioxidants. 

## 2. Results

### 2.1. Growth and Biomass Yield

During the present investigation, the NaCl treatment decreased the length of the shoots, roots and DW by 48.9%, 47.86% and 49.56%, respectively, in comparison to the control. However, GA_3_ and Si individually as well as in combination positively affected plant growth. When treated individually with GA_3_ and Si, the shoot length, root length and DW increased slightly and did not show any significant difference, while the length of the shoots and DW increased by 13.04% and 14.72%, respectively, after combined treatment with GA_3_ and Si over the non-treated (control) plants. Moreover, in comparison to the salt treated *B. juncea* plants, GA_3_ and Si ameliorated the salt stress by enhancing the shoot, root length and DW slightly over NaCl treatment. However, no significant effect was observed individually on the following parameters when treated individually with GA_3_ and Si. Nevertheless, the combined treatment of GA_3_ and Si improved the root length, shoot length and DW by 62.07%, 55.78% and 62.26%, respectively, in NaCl-treated plants (Table 1). 

### 2.2. Photosynthetic Pigments

In the present study, control plants treated with GA_3_ and Si individually and in combination increased Chl *a*, Chl *b*, total Chl and the contents of carotenoid slightly (Table 2). Upon treatment with 200 mM NaCl, we observed a decrease of about 52.66%, 32.39%, 46.67% and 26.53% in Chl *a*, Chl *b*, total chlorophyll and carotenoids contents, respectively, in *B. juncea* plants in comparison to the control. GA_3_ application ameliorated salt (NaCl) stress in *B. juncea* by increasing Chl *a*, Chl *b*, Total Chl and carotenoids by 11.23%, 14.58%, 20.31% and 25%, respectively. Likewise, Si application also increased the Chl *a*, Chl *b*, Total Chl and carotenoids by 40%, 22.19%, 33.59% and 33.33%, respectively. Nevertheless, when the NaCl-stressed plants were treated with GA_3_ and Si in combination the Chl. *a*, Chl.*b*, Total Chl and carotenoids increased by 49.43%, 37.5%. 55.46% and 50%, respectively, and the resultant difference was found to be statistically significant (Table 2).

### 2.3. RWC, Proline, H_2_O_2_, MDA and EL

The NaCl-treated plants showed decreased RWC by 20.94% as compared to the control; however, supplementation of GA_3_ and Si to NaCl-treated plants enhanced the RWC content by 30.48% and 32.89%, respectively. Nevertheless, the combined application of GA3 and Si to NaCl-treated plants showed a maximum increase of 46% in comparison to NaCl-treated plants alone (Table 3).

The proline content was enhanced by 72.13% in NaCl-treated plants as compared to the control. Further enhancement in proline content by 25.03% and 36.9% was observed by the supplementation of GA3 and Si, respectively, over NaCl-treated plants alone. The combined application of GA_3_ and Si to NaCl-treated plants increased the proline content by 45.60% over the salt-treated plants alone and the effect was more pronounced than the individual effect of GA_3_ and Si (Table 3).

NaCl stress increased the H_2_O_2_ content by 219% with respect to the control; however, GA3 and SI decreased the H_2_O_2_ content by 25.20% and 23.30%, respectively, over NaCl- treated plants alone. The combined application of GA_3_ and Si decreased the H_2_O_2_ content by 52.13% in salt-stressed plants and the result was more promising than the individual effect of GA_3_ and Si.

The NaCl-stressed plants showed a 68.03% increase in MDA content as compared to the control. However, the supplementation of GA_3_ and Si alone and in combination (GA_3_ and Si) showed a significant decline by 22.76%, 31.54% and 35.77%, respectively, in MDA content as compared to NaCl-treated plants alone (Table 3).

The EL was increased by 472.48% in NaCl-treated plants as compared to the control. However, when NaCl-stressed plants were treated with GA_3_ and Si, the EL decreased by 46.42% and 51.97%, respectively, over NaCl-treated plants alone. A maximum decrease of 63.73% was observed when NaCl-stressed plants were supplemented with a combination of GA_3_ and Si. The combined effect of GA_3_ and Si was more promising than their individual effect (Table 3).

### 2.4. Effect on Antioxidant Enzyme Activities

**Figure 1:**
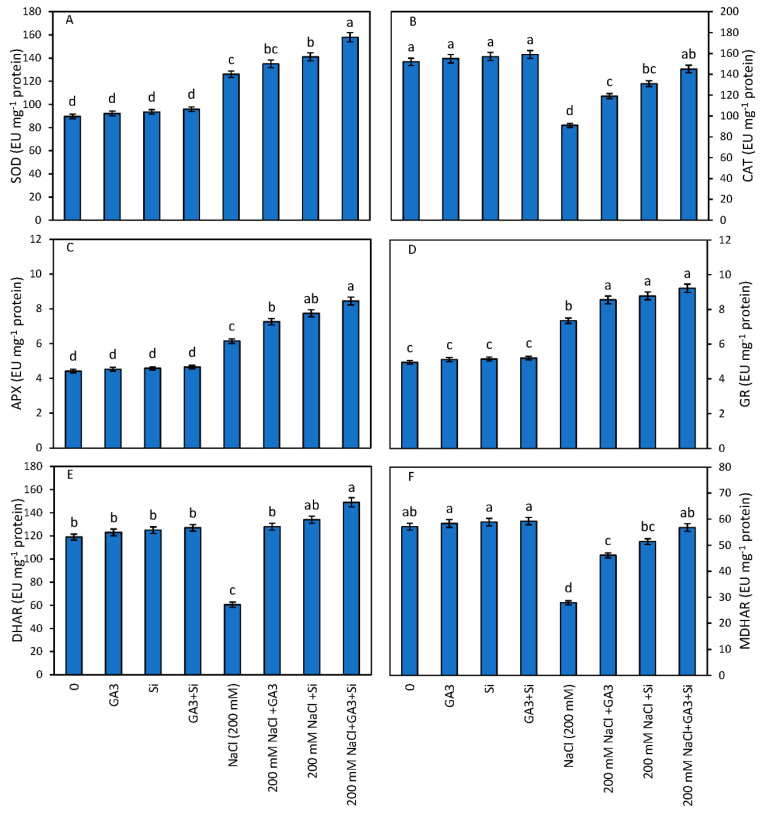
Effect of Gibberellic acid (GA_3_) and silicon (Si) application on (**A**) SOD, (**B**) CAT, (**C**) APX, (**D**) GR, (**E**) DHAR and (**F**). MDHAR of *B. juncea* cultivated under NaCl stress. Mean on the column bar that shares a letter with the other entries is not significantly different at *p* ≤ 0.05 (Bonferroni Test).

The NaCl toxicity enhanced the activity of SOD, APX and GR by 40.45%, 39.23% and 48.48%, respectively as compared to the control. However, the application of GA_3_ alone to NaCl-treated plants further enhanced the SOD, APX and GR activity by 7.14%, 30.76% and 16.32%, respectively, over NaCl-treated plants alone. The supplementation of Si to NaCl-treated plants also enhanced the activity of SOD, APX and GR by 11.90%, 26.05% and 19.45%, respectively over NaCl-treated plants alone. The maximum increase of 25.39%, 37.62% and 25.44% in the activities of SOD, APX and GR was observed when the salt-stressed seedlings were treated in combination (GA_3_ and Si) in comparison to NaCl- treated plants alone (Figure 1).

The seedlings treated with NaCl showed a significant decrease of 40.13% in CAT activity with respect to the control plants. However, NaCl-stressed seedlings supplemented with GA_3_ (NaCl and GA_3_) and Si (NaCl and Si) significantly enhanced the CAT activity by 30.76% and 43.95% as compared to NaCl-treated plants alone. The maximum increase of 59.34% in CAT activity was recorded under the combined application of GA_3_ and Si over NaCl-treated plants alone (Figure 1). 

The control plants supplemented with GA_3_ and Si separately enhanced the DHAR and MDHAR activity by 3.36%, 5.04%, 2.18% and 3.08%, respectively, over the control (Figure 1). The combined supplementation of GA_3_ and Si further enhanced the DHAR activity by 6.72% and MDHAR activity by 3.71% over the control plants. 

The NaCl-treated plants supplied with GA3 and Si separately increased DHAR activity by 111.5% and 121.45% and MDHAR by 65.53% and 84.46%, respectively, over NaCl-treated plants alone. The combined supplementation of GA3 and Si to NaCl- treated plants further enhanced the DHAR and MDHAR by 146.2% and 103.5%, respectively, as compared to NaCl-treated plants alone. (Figure 1). 

### 2.5. AsA-GSH Cycle

**Figure 2:**
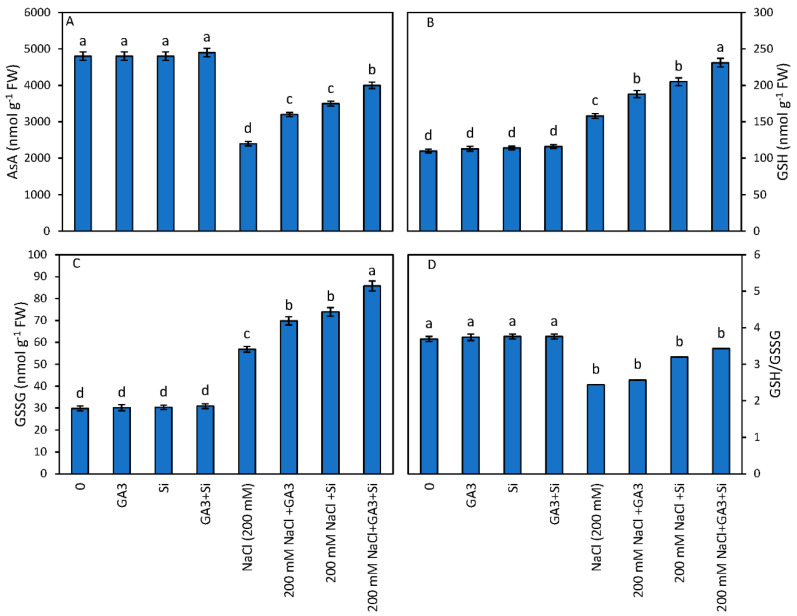
Effect of GA_3_ and Si application on (**A**) AsA, (**B** GSH, (**C**). GSSH and (**D**). GSH/GSSG of *B. juncea* in relation to NaCl stress. Mean on the column bar that shares a letter with the other entries is not significantly different at *p* ≤ 0.05 (Bonferroni Test).

The NaCl toxicity reduced the AsA content by 50% as compared to the control. However, this reduction in AsA was minimised to 33.3% and 27.08% when NaCl stressed plants were treated individually with GA_3_ and Si over NaCl-treated plants alone. The combined GA_3_ and Si supplementation showed a significant increase of 66.6% in AsA content over NaCl-treated plants alone (Figure 2). The application of NaCl resulted in significant increase of 43.6% and 90.8% in the levels of GSH and GSSG content over the control plants. However, the individual treatment of GA_3_ and Si increased the levels of GSH by 18.98% and 29.74% and GSSG levels by 21.9% and 30%, respectively, over NaCl= treated plants alone. The maximum increases of 46.20% and 50.9% were reported when NaCl-treated plants were subjected to combined supplementation of GA_3_ and Si over NaCl-treated plants alone. The NaCl treatment also increased the GSH/GSSG ratio in the present study and the separate application of GA_3_ and Si significantly increased the GSH/GSSG ratio by 5.32% and 31.14% over NaCl-treated plants alone. The maximum increase of 40.57% was reported when GA_3_ and Si was used in combined fashion in NaCl-stressed plants (Figure 2).

### 2.6. Effect on Inorganic Nutrients

The plants exposed to NaCl stress showed a drastic increase in Na concentration by 1149%, but a reduction of 52.55% and 68.8% in K and Ca concentrations were observed in the control plants (Table 4). However, when NaCl-treated plants were supplemented with Si and GA_3_ separately, the concentrations of Na decreased by 45.18%, and 54.21%, the concentration of K increased by 21.18% and 62.47% and Ca by 69.40% and 85.71%, respectively, over NaCl-treated plants alone. The combined application of GA_3_ and Si to NaCl-treated plants lead to a drastic reduction of 74.64% in Na concentration, while an increase of 93.95% and 151.9% in K and Ca concentrations was observed over NaCl-treated plants alone (Table 4). During NaCl stress, the ratio of Na/K increased by 2536.84%; however, after individual and combined application of GA_3_ and Si, this ratio decreased by 54.75%, 71.85% and 86.95% as compared to NaCl-treated plants alone (Table 4).

## 3. Materials and Methods

### 3.1. Layout and Experimental Design

*B. juncea* seeds of the cultivar *varuna* were thoroughly washed and sterilized using sodium hypochlorite, 0.1%. After that, the seeds were allowed to sprout on filter paper with lined petri plates. Thereafter, seedlings were transplanted and raised in Hoagland’s nutrient solution and then shifted in 15 pots of 10 L capacity containing soil with soil physical properties available (P 2.16 mg kg^−1^; SO_4_^2−^ 6.52 mmol L^−1^; Cl^−^ 2.20 mmol L^−1^; K^+^ 0.05 mmol L^−1^; Na^2+^ 3.58; Ca^2+^ + mg^2+^ 3.72; Cu^2+^ 0.28 mg kg^−1^; Cr 0.20 mg kg^−1^; Si 210 mg kg^−1^; pH 7.92; EC 1.75 dS m^−1^ and Soil Organic Carbon (SOC) = 12.80 g kg^−1^). Pots were maintained at a temperature of 25–35 °C and 16 h of light under a glasshouse with 65–75% relative humidity during March and April. Plantlets were supplemented with an analytical grade NaCl (200 mM). Silicon (Si) was supplied in the form of sodium silicate solution (30% SiO_2_ (922587-5G) dissolved in 15% NaOH) in the roots, which were added directly to jars in a dissolved condition. The 1 mg solution of GA_3_ (G7645) was prepared by dissolving 100 mg of GA_3_ in DMSO and then diluting with 80 mL of the aqueous phosphate buffer solution (pH 7.2) in order to achieve good solubility. The freshly prepared GA_3_ solution (75 mL/pot) was spewed onto plants once a week from the starting day (1st) to the end of the experiment (45th) day of the NaCl treatment. Control plants were sprinkled with distilled water and tween-20 (75 mL/pot). Samples were collected after 15 days of treatment with 3 replications in a randomized complete block design. All the chemicals used in the present experiment were purchased from Merck (Rahway, NJ, USA)and Sigma-Aldrich India (Mumbai, India).

### 3.2. Evaluation of Plant Growth Parameters

The root and shoot length were measured with the help of a meter scale and the whole plant height was calculated in cm. The roots were then separated from the shoot, blotted, and subsequently weighed to estimate their fresh weight (FW), then kept in an oven at 80 °C overnight and weighed again to obtain the respective dry weight (DW). The leaf area was calculated by utilizing a portable leaf-area measuring instrument (Systronics 211, Ahmadabad, India), as per the manufacturer’s instructions. Plants were harvested and taken for sampling from each plot.

### 3.3. Leaf Water Content (LWC) and Relative Water Content (RWC)

For the determination of LWC, the fresh weight of the leaves (fresh sample) was measured (FW); for the dry weight measurement (DW), the leaves were dried overnight in an oven at 70 °C and finally LWC was premeditated with equation

LWC = [(FW − DW)/FW] × 100(1)

The fully extended leaves were used for calculating the RWC by using the procedure by Singh et al. [19]. From the interveinal area of the plants, 5 leaf discs were excised, 10 mm in diameter. The fresh weight of the samples (FW) was determined after pooling 20 discs from each replicate. After 4 h of flotation in double-distilled water in petri plates to restore leaf turgidity, the leaves were then weighed again to obtain the turgid weight (TW). The leaf samples were subsequently dehydrated at 80 °C for 48 h to obtain the DW. According to the tests, the leaf discs were completely hydrated in 4 h. The RWC was determined with the help of following formula

RWC (%) = (FW − DW)/(TW − DW) × 100(2)

### 3.4. Evaluation of Chlorophyll and Carotenoid Content

For the estimation of chlorophyll content, dimethyl sulfoxide (DMSO) was used as per the method of Hiscox and Israelstam [20]. Fresh material (100 mg) was inserted inside the test tubes containing DMSO. The tubes were put in an oven for 40 min at 65 °C. A total of 1 mL mixture of aliquot and 2 mL of DMSO was formulated and whirled, followed by absorbance determination spectrophotometrically at 480; 510; 645; and 663 nm (Beckmann 640 D, Fullerton, CA, USA). DMSO was used as a blank. The following equations were used for the calculation of the above-mentioned parameters:

$$\mathrm{Chlorophyll}\text{-}a\;\left(\frac{\mathrm{mg}}{g}\mathrm{fresh}\;\mathrm{wt}.\right)=\frac{12.3_{D663}-\;0.86_{D645}}{d\;\times \;1000\;\times \;W}\;\times \;V$$

$$\mathrm{Chlorophyll}\text{-}b\;\left(\frac{\mathrm{mg}}{g}\mathrm{fresh}\;\mathrm{wt}.\right)=\frac{19.3_{D645}-\;3.60_{D663}}{d\;\times \;1000\;\times \;W}\;\times \;V$$

$$\mathrm{Carotenoids}\;-\left(\frac{\mathrm{mg}}{g}\mathrm{fresh}\;\mathrm{wt}.\right)=\frac{7.6_{\;D480}-\;1.49_{D510}}{d\;\times \;1000\;\times \;W}\;\times \;V$$

$$\mathrm{Total}\;\mathrm{Chlorophyll}\;\left(\frac{\mathrm{mg}}{g}\mathrm{fresh}\;\mathrm{wt}.\right)=\frac{20.2_{D645}+8.02_{D663}}{d\;\times \;1000\;\times \;W}\;\times \;V$$

### 3.5. Electrolytic Leakage (EL)

A total of 20 leaf discs were kept in a boiling test tube with 10 mL of de-ionized water to measure initial electrical conductivity (EC-a). The contents in the test tube were then again heated for 25 min in a water bath at 50 °C and 60 °C, and the EC was measured again (EC-b). Afterwards, the tube contents were once again allowed to boil at 100 °C for 10 min and the EC was recorded again (EC-c) [21]. The EL was then premeditated, as given below:

$$\left(\%\right)\mathrm{Electrolyte}\;\mathrm{leakage}\;=\frac{\mathrm{EC-b}\;-\;\mathrm{EC-a}}{\mathrm{EC-c}}\times 100$$

### 3.6. Proline Determination

The proline content was estimated by Bates et al. [22]. Homogenization of the fresh sample was done by putting 300 mg of sample in 10 mL 3% aq. sulfosalicylic acid and then, centrifugation of the homogenate for 15 min at 10,000× *g*. After that, 2 mL of supernatant aliquot was combined with glacial acetic acid along with ninhydrin in equal volumes and incubated at 100 °C for 1 h. The reaction was completed in an ice bath followed by extraction using 4 mL of toluene, after which the obtained extract was whirled for 20 s. The toluene-containing chromatophore was subsequently extracted from the aqueous phase. The absorbance was noted using a spectrophotometer at 520 nm (Beckmann 640 D, USA) with toluene as a blank.

### 3.7. Determination of Lipid Peroxidation (MDA)

Lipid peroxidation was estimated using the method of Heath and Packer [23]. Homogenization of 300 mg of fresh leaves was done with 2.5 mL 0.1% of trichloroacetic acid (TCA). Afterwards, the homogenate was centrifuged for 10 min at 10,000× *g*. In total, 4 mL 20% of TCA with 0.5% thiobarbituric acid (TBAR) was then added for each 1 mL aliquot. The obtained mixture was allowed to heat at 95 °C for half an hour and was cooled rapidly in an ice bath. The obtained mixture was again centrifuged for 15 min at 10,000× *g*, and the supernatant’s absorbance was taken at 532 nm. These observations were finally modified for undefined turbidity by deducting the absorbance at a wavelength of 600 nm. 

### 3.8. Enzyme Activity Determination

A total of 0.5 g leaves were homogenised in a 50 mM buffer solution of pH 7.0 with 1% polyvinyl pyrrolidine. The centrifugation of homogenate was done at 15,000× *g* at 48 °C for a duration of 20 min. The obtained supernatant was then utilized for the purpose of the enzymatic determination activities of APX, CAT, POD, SOD and GR. 

The method suggested by Bradford [24] was used for estimation of the soluble protein. Bovine serum albumin was used as the standard.

*Superoxide dismutase* (*SOD*) activity was examined by observing its capability to constrain the photo-chemical decrease of NBT [25]. One unit of SOD may be demarcated as the quantity of protein essential for a 50% decline of the SOD-preventable decrease of NBT.

A technique described by Aebi [26] was used in the investigation of the *Catalase (CAT)* activity. The extract containing the enzyme (50 mL) was put into 3 mL 20 mM H_2_O_2_ along with a 50 mM phosphate buffer solution (PBS) of pH 7.0. The reduction in the optical density was observed at 240 nm. The *Glutathione reductase* (*GR*) activity was examined by employing the Rao [27] method. The reacting mixture was constituted of an enzyme extract, 150 mM NADPH and 500 mM glutathione in an oxidized form in a 100 mM buffer solution of pH 7.0 in 1.0 mM EDTA.

*Ascorbate peroxidase* (*APX)* was examined using a spectrophotometer after a reduction in absorbance value at 265 nm [28]. In a 3 mL mixture, 0.1 mM of EDTA, 0.5 mM of ascorbate and 0.1 mM of hydrogen peroxide were combined with 0.1 mL enzyme extract in a 50 mM buffer solution of pH 7.0. The ascorbate oxidation by hydrogen peroxide was accompanied by a decrease in absorbance value at 290 nm. Glutathione in reduced form (GSH) was measured using an enzyme recycling approach [29], where it was oxidised with 5,5’-dithiobis-2-nitrobenzoic acid (DTNB) afterwards, which was reduced with NADPH. Then GSH was derivatized with the help of 2-vinyl-pyridine for measurement of the glutathione in oxidised form (GSSG).

Using a mortar and pestle, 0.5 g of fresh-leaf tissue sample was pulverized in liquid nitrogen. The pulverized tissue was then allowed to suspend in 0.5 mL of 5% sulfosalicylic acid surveyed by centrifugation for 10 min at 12,000× *g*. In total, 300 mL supernatant aliquot was then taken and neutralised with 18 mL of 7.5 M triethanolamine. Then, a sample of 150 mL was utilized to calculate the GSH and GSSG concentrations. Afterwards, a sample was pre-treated for 60 min at 20 °C with 3 mL of 2-vinylpyridine to protect the GSH by making its derivative, for subsequent estimation of GSSG only. In all cases, aliquots of 50 mL from the prepared samples were combined with 700 mL of 0.3 mM NADPH, 100 mL of DTNB, 150 mL of 125 mM SPB (pH 7.0) and 6.3 mM EDTA buffer solution (pH 6.5). After that, an aliquot of 10 mL GR (5 U mL^−1^) was put in the sample, and the variation in the absorbance value was measured at 412 nm at 30 °C. GSH was used to create a standard curve. The reduced (GSH) ratio to oxidised form (GSSG) was used to represent the redox state.

### 3.9. MDHAR and DHAR Activity Determination

The homogenization of 50 mg leaves was done using 1 mL of non-denatured buffer solution containing 0.1 M Tris-HCl (pH 7.5); 0.1 M KCl; 5 mM EDTA; 10 mM DTT; 1 mM PMSF, 0.7 M sucrose and a protease inhibiting cocktail. The semi-liquid mixture was then gestated for 15 min in an ice bath with constant shaking, and then centrifugated at 4 °C for 15 min at 14,000× *g*. The clear supernatant was directly utilized for enzyme analysis activity.

*Mono-dehydroascorbate reductase* (*MDHAR*) was examined by means of a spectrophotometer with the technique defined by Hossain et al. [30]. After that, NADH oxidation-induced reduction in the absorbance at 340 nm was measured. 

*Dehydroascorbate reductase* (*DHAR*) was examined at room temperature by determining the absorbance at 265 nm using the procedure by Nakano and Asada [28]. The increase in absorbance value induced by GSH-dependent generation of ascorbate was monitored spectrophotometrically. The enzyme activity was stated in nmol per mg of protein.

### 3.10. Determination of Ascorbate Content (AsA)

*Ascorbate (AsA*) was examined using an approach defined by Zhang et al. [31]. The AsA value measured was consequently divided into a reduced amount of AsA and a total amount of AsA. Then absorbance was observed at 525 nm.

### 3.11. Determination of Inorganic Cations

The plant samples were wet-ashed using nitric-perchloric acid digestion. Finely ground, oven-dried plant material (0.2 g) was quantitively transferred into 50 mL digestion tubes, to which two microliters of concentrated HNO_3_, followed by 1 mL 70% HClO_4_ was added and finally diluted to 100 mL. Using this mixture, the total cation content of Na^+^, K^+^ and Ca^2+^ was determined by inductively coupled plasma mass spectroscopy (ICPS-7500, Shimadzu). The Na^+^/K^+^ ratio in *B. juncea* leaves was calculated using the corresponding values.

### 3.12. Stastistical Analysis

Statistically, the obtained data was analysed with one-way variance analysis (ANOVA). The values obtained were mean + SD for each group, with five samples of plant growth parameters. *p* values *<* 0.05 were taken as significant using IBM SPSS 26.

## 4. Discussion

Increased salt concentrations in soils owing to agricultural mismanagements is a prime factor that has catastrophic effects on agricultural productivity by decreasing crop growth and survival [32]. Recent studies have demonstrated a substantial decline in crop yields under salinity in wheat [33] and tomato [34]. Hence, a need arises for prevention of crop losses due to increasing soil salinity to fulfil the food requirements of a swelling human population [9,35]. Thus, more research is needed to propose some better choices for strengthening the stress protection in *B. juncea* in order to increase crop productivity. In this regard, we investigated the effectiveness of exogenous applications of GA_3_ and Silicon (Si) in promoting crop development by way of its role in controlling some essential inherent plant characteristics. Excessive concentration of salt in soils may diminish plant morphological characteristics by reducing cell division, resulting in decreased cell elongation and growth because of nutritional imbalance, oxidative and osmotic stress [8]. Silicon dioxide (SiO_2_) is the common form of Si in soil. Solubility of all Si forms is low and biogeochemically immobile. The major soluble forms of Si in the soil are poly- and monosilicic acids; however, monosilicic acid occurs mostly in a feebly adsorbed condition and has low capability to migrate inside the soil. By increasing the monosilicic acid concentration in a soil solution, plants are able to absorb phosphates (P) directly. The amount of monosilicic acid is increased because of the chemical resemblance between phosphate and silicate anions, causing a competitive reaction in the soil. The insolubility of monosilicic acid decreases slightly through interactions with heavy metals, iron, aluminium and manganese [36].

The consequence of negative growth was found to be more prominent in shoots than in roots. The salt stress was more effective by hampering the capability of *B. juncea* plants to captivate soil moisture, leading to stunted growth in plants. An exogenous application of the GA_3_ application modified the shoot, root length and plant DW [5], thus alleviating the adverse impacts of NaCl toxicity by augmenting above mentioned plant traits significantly over non stressed ones [33] (Table 1). GA_3_ was helpful in increasing the length of the roots and shoot in cultivars of oat [5]. Increased levels of GAs such as bioactive GA1 and GA4 contribute to cell elongation as well as to the tolerance to salinity and waterlogging [37]. Similar results were observed by Shaddad and El-Samad [38] upon treatment with GA_3_ in the development of shoots and roots of two cultivars of wheat. The application of GA_3_ in alleviating salinity-related adversities could be due to the activation of some distinct enzymes involved in the synthesis of RNA and proteins [5]. The reduction in root-shoot length and growth progression might be due to NaCl ions toxicity or a decrease in osmotic potential produced by diminished water uptake and nutrient captivation. The impressions of complex salt concentration on root growth and development were more noticeable than the growth of shoots responsible for seedling growth reduction [3]. Ashour [39] reported that an exogenous application of an Si nanoparticle with GA to the *Cupressus macrocarpa* plant improved the root and shoot biomass, plant height, chemical constituents, nutrient uptake and antioxidant system by increasing the indole and phenol contents in the plant.

Si treatment was also responsible for reducing problems caused by water scarcity and increased salt stress by enhancing mineral uptake and transport, thereby increasing growth and yield over non-stressed seedlings [40,41]. In stressful conditions, Si was found to provide mechanical strength to plant tissues, thereby increasing pest resistance, which was the major responsible factor in increasing shoot length, root length and plant DW in *B. juncea.* Enhancement in various plant growth-related characters under different stresses with Si supplementation is well established [42,43].

GA_3_ and silicon both have the ability to alleviate such adverse properties and improve stress resilience, which may be attributed to their critical character in stress reduction [44,45]. In the current study, plant seedlings under salinity stress demonstrated a sharp reduction in various growth-related parameters (Table 1). Reduction in growth under salinity stress may be attributed to ROS production, responsible for biomolecule oxidation, such as nucleic acids, proteins, lipid membranes and enzyme inhibitors [46], and a decrease in photosynthesis [47,48]. A combined application of GA_3_ and Si counteracted the inhibitory effect of salinity stress by enhancing the growth traits of okra [49]. Our results are in corroboration with those of Ahmad et al. [44], who observed that a combined application of GA_3_ and jasmonic acid alleviated cadmium stress in chick peas. The increase in chlorophyll content was found to increase with GA_3_ application in *Sorghum* [3]. Application of Si has also been found to lessen plant salt stress by improving numerous structural, biochemical and physiological attributes [1,50]. It has been reported that GA_3_ application promotes cell proliferation and elongation in several salt stressed plant species [51,52]. Application of GA_3_ to salt-stressed plants improved the stages of cytokinin and IAA in plant leaves, probably owing to the favourable impacts of GA_3_ on ion uptake and plant hormonal homeostasis, as well as photosynthetic development. NaCl stress has been reported to enhance the Na^+^/K^+^ ratio; however, on recovery from stress, the ratio decreased and may be a plant-adaptive response [44,53]. Similarly, Si application may also improve the growth and productivity of salt-stressed *B. juncea* by increasing the photosynthetic rate, improving water levels, decreasing oxidative impairment, regulating osmolytes and increasing antioxidant enzyme activity [47]. Further supplementation of silicate fertilizer improved the vigour and growth progression and hence the yield of cucumber, which also reduced the damage caused by Wilt disease [54].

The detrimental impacts of NaCl on chlorophyll concentrations and carotenoids in plants could be ascribed to the effect of salt concentration on chloroplast structure disorder, increased chloroplast degradation and inhibiting the ribulose-1,5-biphosphate. The decrease in chlorophyll content under NaCl may be because of the repressive effects of several salt ions on the biogenesis of diverse molecules of chlorophyll [3,55]. The momentous intensifications in chl *a*, chl *b* and concentrations of carotenoid in silicon-treated and salinity-stressed plants could be attributed to the ameliorative role of Si in increasing K^+^ and decreasing Na^+^ uptake [56], which in turn promotes photosynthesis, and improves the actions of numerous enzymes, water status and RWC. Our results are consistent with several recent researchers who have demonstrated that Si application decreases Na^+^ and increases potassium uptake under salt and conditions of water deficiency stress in different plants [3,47,50,56]. The constructive impacts of Si on chlorophyll biogenesis and photosynthesis under NaCl toxicity have been reported widely. The application of different heavy metals supplemented with Si enhances the chlorophyll and carotenoid content in the leaves of maize, wheat, sorghum, cucumber and rice [11]. The application of Si-augmented chlorophyll contents under heavy metal stress in different plants was also noted [57,58]. The increase in chlorophyll contents under GA_3_ seed priming, foliar application and combined seed priming and foliar spray was also reported in maize under salinity stress conditions, although the highest increment in the contents of chlorophyll pigments were observed under combined application over the control in stressed seedlings [59]. The present outcomes are in conformity with the preceding results of Yousif et al. [3], who reported an enhancement in leaf chlorophyll and carotenoid contents under GA_3_ application in sorghum. 

Salinity stress adversely affects RWC and the reasons could be the inhibition of water absorption from the soil solution to the plant root system, thereby decreasing the interior supply of water and thus disturbing plant tissues undesirably [47]. Intensification in proline levels under salt stress protects the chlorophyll from its degradation. Moreover, proline as an osmolyte plays an important role in the foraging of harmful ROS, regulating cell redox homeostasis and providing energy [17]. Silicon administration to soybean seedlings alleviated the negative effects of salinity by enhancing endogenous GA_3_ and lowering the ABA level [60], enhanced antioxidant defence system via ROS inhibition, ameliorated photosynthetic activity and relative water status to alleviate the heat stress-induced adversities in date palm [61]. Furthermore, the amelioration of the adverse effects of salt stress with Si application was suggested by increasing bioactive GA (GA1 and GA4) levels, but the levels of other plant hormones declined sharply, which were increased under salinity stress.

A similar effect of increase in RWC and proline were also observed under salt stress due to the exogenous application of Si in mustard, sweet peppers and other plants [47,54,62]. Comparable outcomes of the increase in RWC and proline content by GA_3_ supplementation were also reported by Yousif et al. [3], Chauhan et al. [5] and Tuna et al. [17]. In the present study, we examined the impact of Si and GA_3_ on EL, lipid peroxidation (MDA) and H_2_O_2_ content under salt stress This significant increase in EL, MDA and H_2_O_2_ content under salt stress was also observed by Kaya et al. [55]. The deleterious effects of H_2_O_2_ on plant membrane leads to lipid peroxidation and an electrolyte leakage [17,47]. This intensification in MDA may probably be due to the oxidative stress in chloroplasts and mitochondria, thereby enhancing MDA content [53]. Furthermore, an application of Si overcomes these damaging effects of salt stress, including a significant decrease in EL, MDA and H_2_O_2_ levels. This can be attributed to the fact that Si has a significant role in providing stability to plasma membranes and increasing the concentration of osmolyte amassing, which results in ROS foraging, particularly H_2_O_2_. Similar results of the decrease in MDA, EL and H_2_O_2_ under Si application in salinity stress were also observed in sweet peppers and sorghum [3,47]. Further, our results demonstrating the decrease in MDA under GA_3_ application in maize plants are in line with the outcomes of Tuna et al. [17], who observed decreased activity in MDA by increasing the exogenous application of GA_3_ under salinity stress.

Our results of the enhanced activity of CAT, SOD, GR, APX, DHAR and MDHAR under salt stress is corroborated by the findings of other researchers [3,15,43,54]. A similar increase in activities of different antioxidants under salt stress were also observed by Yousif et al. [3]., Tuna et al. [17], Abdelaal et al. [47] and Shahzad et al. [59]. Exogenous application of Si and GA_3_ under salinity stress amplified the levels of various antioxidant enzymes. Si application in *Oryza sativa* plants under salt stress reduced the accumulation of MDA and enhanced the activity of enzymatic antioxidants such as CAT, POD and PPO [11]. Torabi et al. [63] are of the opinion that Si application to borage plants decreased the activities of SOD significantly. Nevertheless, Shekari et al. [7] found that the activities of CAT, APX, SOD and POD were extremely amplified upon Si application to herbal *Anethum graveolens* plants under NaCl stress. Similar patterns of SOD, GPX, APX, GR and CAT activities were also detected by Al-aghabary et al. [64], Liang et al. [65] and Zhu et al. [66]. Furthermore, exogenous Si application was also related to the upregulating of the activities of antioxidant enzyme activities of APX, MDHAR, DHAR, GR, GST, GPX and CAT in rape seedlings [67]. Under salinity stress, the exogenous supplementation of GA_3_ enhanced the expression of different antioxidants in sweet peppers [59]. Comparable results have been previously described by Saeidi-Sar et al. [6]. However, externally applied GA_3_ was found to increase the activities of MDHAR and DHAR under water stress in spring wheat [16]. The observed increase in the aforementioned antioxidants was noticed under exogenous application of individual and combined jasmonic acid and GA_3_ under Cd toxicity [44].

Ascorbate is one of the most important and effective antioxidants among antioxidants providing protection to plant cells under oxidative stress. This antioxidant works in the AsA-GSH cycle organized with GSH [68]. The levels of AsA decreased under salt stress in the present study coincides with the findings in other studies [67,68,69]. This reduction in AsA was accountable for increasing H_2_O_2,_ leading to oxidative stress. Due to the activities of DHA and MDHAR enzymes, AsA can be renewed through the AsA-GSH cycle [68]. The increase in AsA in Si-treated seedlings was accompanied by increased levels of DHAR and MDHAR activities and in those that regenerated AsA effectively. A salinity-induced increase in GSH and GSSG in in the current study is in line with the results of Hasanuzzaman et al. [67] and Nahar et al. [69]. GSH reduces oxidation stress by scavenging various ROS species after being involved in the process of ROS detoxification. GSG is transmuted to GSSG, for which the level of GSSG is also amplified under salinity stress, which is an indication of stress. Glutathione reductase helps to recover GSH, which disturbs the GSH pool and the ratio of GSH/GSSG in plants to some extent (Figure 2). The contents of GSH in plants is also controlled by GSH biosynthetic enzyme pathways [70]. In other words, stress-tolerant plants instantly recycle GSSG more effectively into GSH so that plants become ready to purify ROS once more. The levels of GSH and GSSG become much improved after the addition of Si, and the reason for this is increased GSH and GSSG levels due to the improved activities of GR. Exogenous Si application was helpful in increasing AsA and GSH levels in sunflowers under salinity stress [71]. Silica uptake in large amounts in rice plants was successful in enhancing the activities of different antioxidants under salinity stress [72]. In a similar manner, the exogenic application of Si rises GSH content, and the activities of APX and MDHAR in sorghum raised under salt stress [73]. The enhanced actions of three important enzymes of the AsA-GSH cycle, i.e., APX, MDHAR and GR, coupled with an increase in activities of AsA and GSH with reducing oxidative stress, was observed in Si supplemented cucumber suffering from chilling-induced oxidative stress [74]. Similar results of an increase in levels of GSH and GSSG were reported in rape seedlings under the addition of Si to alleviate salt stress, as reported by Hasanuzzaman et al. [67]. An increase in concentration of GSH and GSSG under GA_3_ and jasmonates has also been reported in *Artemisia absinthium* L. [75]. The reported increase of AsA and GSH in the leaf, root and stem under GA_3_ treatment in *C. roseus* were reported by Jaleel et al. [18], and the maximum increase was reported in the leaves. The increase in levels of AsA, GSH and GSSG were found to increase through the supplementation of GA^3^ under herbicides and drought stress [76].

The salinity stressed *B. juncea* plant seedlings showed a significant decrease in sodium, potassium calcium and N:K ratio in comparison to the control treatment (Table 4). However, exogenic Si and GA_3_ application separately and in amalgamation increased the plant nutrients Na, K, Ca and N:K ratio following Si application under NaCl stress. The helping role of Si in mineral nutrition and uptake of different nutrients were reported in grapes [77], sweet peppers [47] and maize [78]. This role of Si can be attributed to declining levels of Na^+^ and enhancing K^+^ content [79]. GA_3_ application improved shoot calcium concentration in the tissues of the SARC-1 seed cultivars of wheat [80]. This may be the reason behind why the permeability of the membrane was distorted in *B. juncea* salt stressed leaves, where levels of calcium were very low and its enhancement under GA_3_ restored the integrity of cell membranes in NaCl-stressed plants. A higher concentration of Ca^2+^ in plants was helpful in attaining healthier crop subsistence with greater plant vigour and development under stressful environmental conditions.

NaCl toxicity causes a noteworthy reduction in K^+^ uptake, although exogenous application of GA_3_-enhanced K^+^ uptake and the results coincide with the findings of Tuna et al. [17], Kaya et al. [55] and Ashraf et al. [80] in maize and wheat, respectively. It has a wide acceptance that the rivalry between Na^+^ and K^+^ leads to the decreased levels of interior K^+^ at higher concentrations of NaCl [55], which results in high ratios of Na:K and reduces plant growth. Interestingly, there lies a synergistic relationship between Si and GAs in mediating several adaptive responses to counteract detrimental stress conditions. Silicon treatment significantly influences endogenous levels of GA, especially during unfavorable conditions. For instance, Si has been shown to up-regulate the production of endogenous bioactive GAs in response to salinity stress [60,81].

It is essential to understand how underground plant roots are skilled to distinguish between indispensable K^+^ and lethal Na^+^ ions. This difference lies in the fact that similar ionic radius and ion hydration energies are the two essential issues that determine how K^+^ and Na+ ions transfer over membrane proteins into cells and to the whole plant [17]. Additional study on the conceivable mechanism of Si and GA_3_ uptake and conveyance in *B. juncea* individually and in combined application is of extreme importance to decipher Si- and GA_3_-induced beneficial effects in alleviating salt stress, which needs more research in the future.

## 5. Conclusions

From the current results, it was observed that NaCl decreased plant biomass, photosynthetic pigments and RWC and enhanced the production of ROS, notably H_2_O_2_, which in turn enhanced lipid peroxidation and electrolyte leakage. The mineral uptake and Na/K ratio are also disturbed by the NaCl toxicity. However, the supplementation of GA_3_ and Si individually as well as in combination showed the mitigating role through the modulation of physio-biochemical attributes and enhanced activities of enzymatic and non-enzymatic antioxidants. The combined effect of GA3 and Si was more effective in raising the tolerance against NaCl toxicity than through their individual effects. The present study will be useful for bringing marginal land under cultivation through this approach.

## Figures and Tables

**Table 1:** Result of gibberellic acid (GA_3_) and silicon (Si) application on shoot length, root length and dry weight of *B. juncea* cultivated under NaCl salt stress.

Treatments	Shoot Length (cm)	Root Length (cm)	Dry Weight (g Plant^−1^)
0	45.32 ± 1.05 ^b^	18.74 ± 0.44 ^a^	12.77 ± 0.3 ^b^
GA_3_	47.11 ± 1.22 ^ab^	19.11 ± 0.5 ^a^	13.24 ± 0.35 ^ab^
Si	48.42 ± 1.22 ^ab^	19.87 ± 0.5 ^a^	13.89 ± 0.35 ^ab^
GA_3_ + Si	51.23 ± 1.3 ^a^	20.45 ± 0.51 ^a^	14.65 ± 0.37 ^a^
NaCl (200 mM)	23.15 ± 0.63 ^e^	9.77 ± 0.25 ^d^	6.44 ± 0.18 ^e^
200 mM NaCl +GA_3_	30.55 ± 0.63 ^d^	12.83 ± 0.26 ^c^	8.23 ± 0.18 ^d^
200 mM NaCl +Si	31.62 ± 0.66 ^d^	13.77 ± 0.28 ^c^	8.89 ± 0.19 ^d^
200 mM NaCl + GA_3_ + Si	37.52 ± 0.84 ^c^	15.22 ± 0.34 ^b^	10.45 ± 0.24 ^c^

Mean in the column that shares a letter with the other entries is not significantly different at *p* ≤ 0.05 (Bonferroni Test).

**Table 2:** Impact of gibberellic acid (GA_3_) and silicon (Si) application on pigments of *B. juncea* cultivated under NaCl stress.

Treatments	Chl. *a* (mg g^−1^ FW)	Chl. *b* (mg g^−1^ FW)	Total Chlorophyll (mg g^−1^ FW)	Carotenoids (mg g^−1^ FW)
0	1.69 ± 0.04 ^a^	0.71 ± 0.02 ^bc^	2.4 ± 0.06 ^a^	0.49 ± 0.01 ^abc^
GA_3_	1.75 ± 0.05 ^a^	0.75 ± 0.02 ^ab^	2.5 ± 0.06 ^a^	0.52 ± 0.01 ^ab^
Si	1.77 ± 0.05 ^a^	0.79 ± 0.02 ^ab^	2.56 ± 0.06 ^a^	0.53 ± 0.01 ^ab^
GA_3_ + Si	1.82 ± 0.05 ^a^	0.83 ± 0.02 ^a^	2.65 ± 0.07 ^a^	0.54 ± 0.01 ^a^
NaCl (200 mM)	0.89 ± 0.03 ^d^	0.48 ± 0.01 ^f^	1.28 ± 0.03 ^d^	0.36 ± 0.01 ^d^
200 mM NaCl +GA_3_	0.99 ± 0.02 ^cd^	0.55 ± 0.01 ^ef^	1.54 ± 0.03 ^cd^	0.45 ± 0.01 ^c^
200 mM NaCl +Si	1.12 ± 0.02 ^c^	0.59 ± 0.01 ^de^	1.71 ± 0.04 ^c^	0.48 ± 0.01 ^bc^
200 mMNaCl + GA_3_ + Si	1.33 ± 0.03 ^b^	0.66 ± 0.01 ^cd^	1.99 ± 0.05 ^b^	0.54 ± 0.01 ^a^

Mean in the column that shares a letter with the other entries is not significantly different at *p* ≤ 0.05 (Bonferroni Test).

**Table 3:** Outcome of gibberellic acid (GA_3_) and silicon (Si) application on RWC, proline, H_2_O_2_, MDA and electrolyte leakage of *B. juncea* cultivated under NaCl stress.

Treatments	RWC%	Proline (µg g^−1^ FW)	H_2_O_2_ (nmol g^−1^ FW)	MDA (µg g^−1^ FW)	Electrolyte Leakage %
0	91.77 ± 2.19 ^a^	59.11 ± 2.02 ^d^	8.22 ± 0.25 ^d^	3.66 ± 0.08 ^d^	11.45 ± 0.5 ^d^
GA_3_	92.85 ± 2.36 ^a^	62.15 ± 2.16 ^d^	7.45 ± 0.48 ^d^	3.59 ± 0.08 ^d^	10.98 ± 1.47 ^d^
Si	92.8 ± 2.26 ^a^	63 ± 1.69 ^d^	7.11 ± 0.31 ^d^	3.55 ± 0.07 ^d^	10.55 ± 0.63 ^d^
GA_3_ + Si	94.11 ± 2.29 ^a^	63.81 ± 1.93 ^d^	7.18 ± 0.27 ^d^	3.49 ± 0.07 ^d^	9.73 ± 0.55 ^d^
NaCl (200 mM)	55.6 ± 1.19 ^c^	101.75 ± 2.24 ^c^	26.22 ± 0.72 ^a^	6.15 ± 0.16 ^a^	65.55 ± 1.83 ^a^
200 mM NaCl +GA_3_	72.55 ± 1.47 ^b^	127.22 ± 3.31 ^b^	19.61 ± 0.53 ^b^	4.75 ± 0.12 ^b^	35.27 ± 0.96 ^b^
200 mM NaCl +Si	76.89 ± 1.5 ^b^	139.34 ± 3.68 ^ab^	17.75 ± 0.47 ^b^	4.21 ± 0.1 ^c^	31.48 ± 0.85 ^b^
200 mM NaCl + GA_3_ + Si	81.22 ± 1.88 ^b^	148.15 ± 3.92 ^a^	12.55 ± 0.36 ^c^	3.95 ± 0.08 ^cd^	23.77 ± 1.05 ^c^

Mean in the column that shares a letter with the other entries is not significantly different at *p* ≤ 0.05 (Bonferroni Test).

**Table 4:** Effect of gibberellic acid and silicon application on Na, K, Na/K and Ca of *B. juncea* cultivated under NaCl stress.

Treatments	Na (mg g^−1^ DW)	K (mg g^−1^ DW)	Na/K	Ca (mg g^−1^ DW)
0	2.01 ± 0.25 ^d^	35.22 ± 0.83 ^ab^	0.06 ± 0.01 ^e^	4.95 ± 0.12 ^a^
GA_3_	2.12 ± 0.64 ^d^	36.12 ± 0.95 ^ab^	0.06 ± 0.02 ^e^	5.12 ± 0.14 ^a^
Si	2.14 ± 0.31 ^d^	36.52 ± 0.94 ^a^	0.06 ± 0.01 ^e^	5.25 ± 0.14 ^a^
GA_3_ + Si	2.15 ± 0.25 ^d^	37.65 ± 0.93 ^a^	0.06 ± 0.01 ^e^	5.39 ± 0.14 ^a^
NaCl (200 mM)	25.12 ± 0.71 ^a^	16.71 ± 0.5 ^d^	1.51 ± 0.06 ^a^	1.54 ± 0.09 ^d^
200 mM NaCl +GA_3_	13.77 ± 0.39 ^b^	20.25 ± 0.47 ^d^	0.68 ± 0.02 ^b^	2.61 ± 0.07 ^c^
200 mM NaCl +Si	11.5 ± 0.32 ^b^	27.15 ± 0.54 ^c^	0.42 ± 0.01 ^c^	2.86 ± 0.07 ^c^
200 mM NaCl + GA_3_ + Si	6.37 ± 0.48 ^c^	32.41 ± 0.79 ^b^	0.2 ± 0.02 ^d^	3.88 ± 0.09 ^b^

Mean in the column that shares a letter with the other entries is not significantly different at *p* ≤ 0.05 (Bonferroni Test).

## Data Availability

The data is available in the manuscript.
